# Slit1 Promotes Hypertrophic Scar Formation Through the TGF-β Signaling Pathway

**DOI:** 10.3390/medicina60122051

**Published:** 2024-12-12

**Authors:** Hui Song Cui, Ya Xin Zheng, Yoon Soo Cho, Yu Mi Ro, Kibum Jeon, So Young Joo, Cheong Hoon Seo

**Affiliations:** 1 Burn Institute, Hangang Sacred Heart Hospital, College of Medicine, Hallym University, 94-200 Yeongdeungpo-Dong, Yeongdeungpo-Ku, Seoul 07247, Republic of Korea; bioeast007@naver.com (H.S.C.); yxzheng2023@gmail.com (Y.X.Z.); nym8060@hanmail.net (Y.M.R.); 2 Department of Rehabilitation Medicine, Hangang Sacred Heart Hospital, College of Medicine, Hallym University, 94-200 Yeongdeungpo-Dong, Yeongdeungpo-Ku, Seoul 07247, Republic of Korea; hamays@hanmail.net; 3 Department of Laboratory Medicine, Hangang Sacred Heart Hospital, College of Medicine, Hallym University, 94-200 Yeongdeungpo-Dong, Yeongdeungpo-Ku, Seoul 07247, Republic of Korea; pourmythe@naver.com

**Keywords:** Slit1, post-burn hypertrophic scar, fibroblast

## Abstract

*Background and objectives:* Slit1 is a secreted protein that is closely related to cell movement and adhesion. Few studies related to fibrosis exist, and the preponderance of current research is confined to the proliferation and differentiation of neural systems. Hypertrophic scars (HTSs) are delineated by an overproduction of the extracellular matrix (ECM) by activated fibroblasts, leading to anomalous fibrosis, which is a severe sequela of burns. However, the functionality of Slit1 in HTS formation remains unknown. We aimed to investigate whether Slit1 regulates fibroblasts through a fibrosis-related mechanism derived from post-burn HTS tissues and normal patient tissues. *Methods:* Human normal fibroblasts (HNFs) and hypertrophic scar fibroblasts (HTSFs) were extracted from normal skin and post-burn HTS tissues, with settings grouped according to the patient of origin. Cell proliferation was evaluated using a CellTiter-Glo Luminescent Cell Viability Assay Kit. Cell migration experiments were carried out using a μ-Dish insert system. Protein and mRNA expression levels were quantified by Western blot and quantitative real-time polymerase chain reaction. *Results:* We found increased expressions of Slit1 in HTS tissues and HTSFs compared to normal tissues and HNFs. The treatment of human recombinant Slit1 protein (rSlit1) within HNFs promoted cell proliferation and differentiation, leading to an upregulation in ECM components such as α-SMA, type I and III collagen, and fibronectin. The treatment of rSlit1 in HNFs facilitated cell migration, concurrent with enhanced levels of N-cadherin and vimentin, and a diminished expression of E-cadherin. Treatment with rSlit1 resulted in the phosphorylation of SMAD pathway proteins, including SMAD2, SMAD3, and SMAD1/5/8, and non-SMAD pathway proteins, including TAK1, JNK1, ERK1/2, and p38, in HNFs. *Conclusions:* Exogenous Slit1 potentiates the epithelial–mesenchymal transition and upregulates SMAD and non-SMAD signaling pathways in HNFs, leading to the development of HTS, suggesting that Slit1 is a promising new target for the treatment of post-burn HTS.

## 1. Introduction

Hypertrophic scars (HTSs) are recognized as a type of abnormal scarring, and their etiology is primarily attributed to external stimuli, among which burns exhibit the highest incidence [1]. HTSs are often associated with sequelae such as pruritus and pain and present an unesthetic appearance due to tissue contracture, discoloration, or protrusion. Consequently, HTSs impose numerous inconveniences on patients’ lives and may trigger negative psychological impacts, such as depression and anxiety [2].

Fibroblasts are crucial for the development of HTS after burn injuries. Fibroblasts proliferate and differentiate into myofibroblasts when stimulated by cytokines or growth factors, which are characterized by the expression of α-smooth muscle actin (αSMA) to generate contractile forces within skin that facilitate wound closure [3]. Fibroblasts secrete a fibronectin matrix and subsequently produce typical fibrotic collagens, including types I and III, which provisionally undergo initial crosslinking to form the extracellular matrix (ECM) [4]. However, overactivated fibroblasts can produce excess ECM, resulting in HTS.

Numerous molecular signaling pathways are involved in HTS formation. Among them, the classical transforming growth factor beta (TGF-β)-mediated drosophila mothers against decapentaplegic protein (SMAD) pathway is a primary mediator of fibrosis [5]. Phosphorylated TGF-β1 ligands, comprising transforming growth factor-beta type 1 receptor (TβR1) and type 2 receptor (TβR2), facilitate the activation of intracellular receptor-regulated SMAD (R-SMAD). R-SMADs interact with their common partner SMAD (co-SMADs) to facilitate their translocation into the nucleus, where they are essential to regulate gene transcription [5,6]. Conversely, TGF-β can also mediate signal transduction through SMAD-independent pathways, such as the activation of the extracellular signal-regulated kinase (ERK)/mitogen-activated protein kinase (MAPK) pathway and transforming growth factor-β-activated kinase 1 (TAK1) in conjunction with downstream signaling p38 MAPK and c-Jun N-terminal kinase (JNK). Concurrently, these protein kinases modulate the activity of R-SMAD or co-SMAD, thereby influencing the classical SMAD pathway [7].

Despite the ongoing research, the underlying causes of HTS remain unclear. Therefore, it is necessary to develop effective therapeutic approaches to prevent scarring. Scar revision can only be accomplished through surgical intervention or by employing pharmacological strategies designed to reduce the HTS [8].

The Slit family comprises secreted glycoproteins that regulate cellular physiological activities by binding to Robo receptors. There are three distinct types of Slit proteins (Slit1, Slit2, and Slit3) and four types of Robo receptors (Robo1, Robo2, Robo3, and Robo4) in vertebrates [9]. The Slit/Robo signaling pathway is involved in cell proliferation, chemotaxis of inflammatory cells, tumor cell migration, and fibrosis [9]. Recently, the Slit family has emerged as an entry point in fibrosis research, with Slit2 being the most extensively studied. For instance, the Slit2–Robo1 signaling pathway facilitates fibrotic processes within the liver [10] and heart [11]. Slit3 also contributes to the regulation of fibrotic collagen synthesis via receptor Robo1 [12]. To date, the literature concerning Slit1 in fibrosis research is significantly limited compared to that on Slit2 and Slit3, and its role in post-burn HTS reagents is largely uninvestigated.

In preliminary experiments, we observed an elevated expression of Slit1 in both HTS tissues and hypertrophic scar fibroblasts (HTSFs) compared to normal tissues and human normal fibroblasts (HNFs). Consequently, we suggest that Slit1 may serve as a critical determinant in the pathogenesis of post-burn HTS. According to this investigation, HNFs will be cultured with human recombinant Slit1 (rSlit1), and the expression of fibrosis markers will be evaluated to investigate the potential promoting effect of Slit1 on scar formation. This study aimed to clarify the involvement of Slit1 in HTS formation through the SMAD and non-SMAD pathways, thereby providing novel therapeutic targets for the prevention and treatment of post-burn HTS.

## 2. Materials and Methods

### 2.1. Patients and Samples

To mitigate errors arising from individual variability, HTS and normal skin tissues were paired as an experimental group according to the patient source. Samples were collected from patients undergoing surgical treatment, all of whom provided written informed consent. Table 1 shows the demographic and clinical information of the patients. All experimental protocols of this study were approved by Institutional Review Board of Hallym University Hangang Sacred Heart Hospital and were carried out in compliance with the guidelines (Hallym University Hangang Sacred Heart Hospital Institutional Review Board, registration number 2023-022).

### 2.2. Histopathology

The matched fresh normal skin and HTS tissues were immobilized in 4% neutral buffered formalin (Biosesang, Seongnam, Republic of Korea) and maintained for 72 h at 25 °C. Subsequently, the tissues were dehydrated in sequential concentrations of 50%, 70%, 80%, 90%, 95%, and 100% ethanol (Merck Millipore, Billerica, MA, USA), clarified with benzene (Dunsan pure chemicals, Ansan, Republic of Korea), and embedded in paraffin blocks. Paraffin tissue sections were cut into slices with a thickness of 4 μm and mounted on silane-coated slides (Muto Pure Chemicals, Tokyo, Japan). The sections were subjected to deparaffinization, rehydration, stained with hematoxylin and eosin, respectively, and covered with mounting medium (Dako, Santa Clara, CA, USA). Images were captured using a Leica DM 750 microscope equipped with an ICC 50 HD camera (Leica Microsystems GmbH, Wetzlar, Germany).

### 2.3. Fibroblasts Isolation and Culture

All procedures involving fibroblasts were performed as described previously [13]. After three sequential rinses with ethanol, followed by three rinses with cold Dulbecco’s phosphate-buffered saline (DPBS) (Biowest, Riverside, MO, USA), skin samples were sectioned into fragments approximately 1 mm^2^ in size. Tissue fragments were incubated in dispase II solution (1 unit/mL; Gibco, Waltham, MA, USA) at 4 °C for 16 h. After this, the dermal tissue was carefully isolated and transferred to a collagenase type IV solution (500 units/mL, Gibco, Waltham, MA, USA) for enzymatic digestion at 37 °C for 1 h. The cellular deposit was obtained by centrifugation at 1200 rpm for 5 min, and subsequently redistributed in medium within T75 flasks (Eppendorf, Hamburg, Germany) for incubation in a humid environment with 5% CO_2_ at 37 °C. The culture medium consisted of high-glucose Dulbecco’s modified Eagle’s medium supplemented with 10% fetal bovine serum (FBS) (Biowest, Riverside, MO, USA) and 1% antibiotic–antimycotic solution containing penicillin, streptomycin, and amphotericin B (Gibco, Waltham, MA, USA). The expanded HNFs and HTSFs were used in the second passage. 

### 2.4. Recombinant Slit1 Protein Treatment

When the density of HNFs reached 50% to 60%, the concentration of FBS was adjusted to 0.05% for a 24 h starvation period. HNFs were treated with rSlit1 (Novus Biologicals, R&D Systems, Centennial, CO, USA, Catalog 6514) at concentrations of 1, 10, and 100 ng/mL. The control group was treated with DPBS. All groups were cultured for either 2 or 24 h based on the experimental requirements.

### 2.5. Cell Proliferation Assay

Cell proliferation was evaluated using the CellTiter-Glo Luminescent Cell Viability Assay Kit (Promega, Madison, WI, USA). HNFs were inoculated into a 96-well plate at a density of 1 × 10^4^ cells/well. Following a 48 h culture period and an additional 24 h of starvation, the HNFs were treated with rSlit1 at three concentrations for another 48 h. The control group was treated with DPBS, whereas the blanks were maintained solely in the medium. We dispensed 100 μL of CellTiter-Glo reagent to every well holding 100 μL of medium, and incubated the solution for 2 h at 37 °C. The luminescence was assessed using a DTX 880 multimode detector (Beckman Coulter, Fullerton, CA, USA) at an excitation wavelength of 490 nm. The following formula × 100% was used to assess cell viability:

$$\frac{\mathrm{sample}\;\mathrm{luminescence}\;-\;\mathrm{background}\;\mathrm{luminescence}}{\mathrm{control}\;\mathrm{sample}\;\mathrm{luminescence}\;-\;\mathrm{background}\;\mathrm{luminescence}}$$

### 2.6. Cell Migration Assay

Cell migration was evaluated through a 2-well culture-insert system in μ-dishes (Ibidi, GmbH, Planegg, Germany), in accordance with the instructions delineated earlier [13]. HNFs were cultured at identical densities and subjected to the same procedural conditions of cell culture, starvation, and rSlit1 treatment, as described above, for cell proliferation. Light microscopy (IX 70, Olympus, Tokyo, Japan) was used to capture images at both 0 and 48 h, and the images were subsequently analyzed using Image J software (version 1.53t, National Institutes of Health, Bethesda, MD, USA). The control group treated with DPBS was used as a reference for normalizing migration to 100%.

### 2.7. Reverse Transcription–Quantitative Polymerase Chain Reaction (RT-qPCR)

HNFs were collected by detaching with Accutase^®^ solution (Thermo Fisher Scientific, Waltham, MA, USA). Total RNA was extracted utilizing RNAzol (Cancer Rop Co., Seoul, Republic of Korea) and ReliaPrep™ RNA Miniprep System kit (Promega, Madison, WI, USA), and its concentration was quantified with a Nanodrop spectrophotometer (BioTek, Winooski, VT, USA), in accordance with correspondent introductions from manufacturers. Isolated RNA was utilized for cDNA synthesis through the PrimeScript™ RT Master Mix (Perfect Real Time) (Takara, Shiga, Japan). The qRT-PCR system consisted of cDNA, 2 × PCR premix (Takara, Shiga, Japan), and specific primers (Table 2) for execution on a LightCycler 96 system (Roche, Basel, Switzerland). The quantification of data was performed through the 2^−ΔΔCT^ method, with GAPDH serving as a reference.

### 2.8. Western Blot

Proteins from HNFs and HSFs were isolated by cell harvesting, followed by extraction with RIPA buffer supplemented with protease and phosphatase inhibitors (Sigma-Aldrich, St. Louis, MO, USA). After determining protein concentrations with BCA kit (Thermo Fisher Scientific), the samples were admixed with 5× SDS-PAGE protein loading buffer (Cell Signaling Technology, Danvers, MA, USA) and heated at 95 °C for 3 min before being stored at −80 °C. For electrophoresis, 20 μg of protein were loaded into each well, followed by electrotransfer onto polyvinylidene difluoride (PVDF) membranes (MilliporeSigma, Burlington, MA, USA). The membranes were incubated with 5% skim milk at room temperature for a duration of 1 h to reduce non-specific binding; subsequently, primary antibodies were incubated overnight at 4 °C as described in Table 3. Following three washes with 1× TBST buffer (Tris-buffered saline with Tween-20; Sigma-Aldrich, St. Louis, MO, USA), the membranes were treated with secondary antibodies conjugated to horseradish peroxidase (HRP) (Jackson ImmunoResearch Laboratories, West Grove, PA, USA) at room temperature for 2 h, specifically goat anti-rabbit or goat anti-mouse IgG (Merck Millipore, Billerica, MA, USA). After three washes with 1× TBST, the membranes were developed, and bands were visualized using a chemiluminescence imaging system (WSE-6100, ATTO, Tokyo, Japan). Image analysis was conducted using CS Analyzer 4 software (ATTO Corporation, Tokyo, Japan), with normalization relative to the levels of β-actin or GAPDH (ATTO, Tokyo, Japan).

### 2.9. Statistical Analysis

All collected data are represented as mean ± standard deviation (SD) for statistical analysis using PASW Statistics 24 (SPSS Inc., Chicago, IL, USA). Comparative analyses between the experimental and control groups were conducted using Kruskal–Wallis one-way analysis of variance and Mann–Whitney U tests. Statistical significance was defined as a *p* value < 0.05 or 0.01.

## 3. Results

### 3.1. Slit1 Expression Levels Were Higher in HTSFs

**Figure 1:**
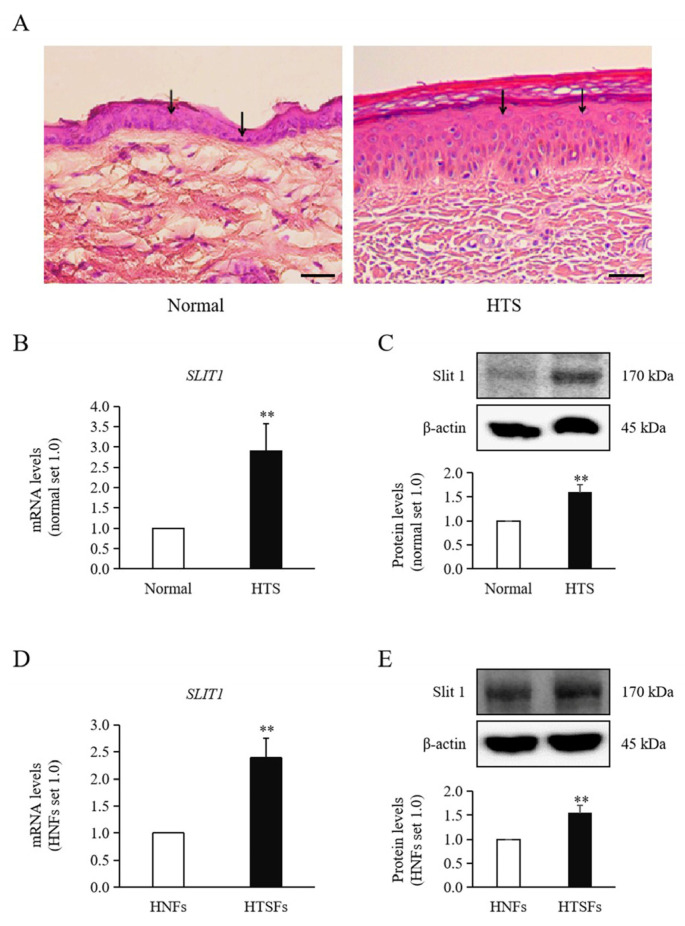
Tissue morphology and expression of Slit1 in tissues and fibroblasts. (**A**) H&E staining in normal skin and hypertrophic scar (HTS). The thickness of the epidermis in HTS appears to be greater than that of normal skin. The arrow marked out the epithelial layer of tissue. Images were acquired at ×10 magnification, scale bar = 50 μm. (**B**,**C**) Significantly increased levels of both mRNA and protein of Slit1 were observed in HTS tissue compared to those in normal tissues. ** *p* < 0.01, vs. Normal. (**D**,**E**) Significantly increased levels of both mRNA and protein of Slit1 were observed in HTSFs compared to those in HNFs. HNFs and HTSFs were extracted from normal skin tissues and post-burn HTS tissues obtained from the same patients. ** *p* < 0.01, vs. HNFs. Data represent the mean ± SD; n = 3.

Histomorphological analysis using H&E staining revealed an increase in epidermal thickness of HTS tissue as compared to the normal (Figure 1A). Quantitative RT-PCR and Western blotting were performed to measure Slit1 expression at the mRNA and protein levels, respectively, in HTS and normal skin tissues, as well as in HTSFs and HNFs from patients with burn injury. The expression of Slit1 within HTS tissue was significantly higher in comparison with those in normal tissue (mRNA: 2.90 ± 0.37-fold increase, protein: 1.58 ± 0.17-fold increase; *p* < 0.01, Figure 1B,C), and analogously, that in HTSFs manifested significantly less than HNFs (mRNA: 2.39 ± 0.36-fold increase, protein: 1.53 ± 0.18-fold increase; *p* < 0.01, Figure 1D,E).

### 3.2. Recombinant Slit1-Induced Cell Proliferation and Differentiation in HNFs

**Figure 2:**
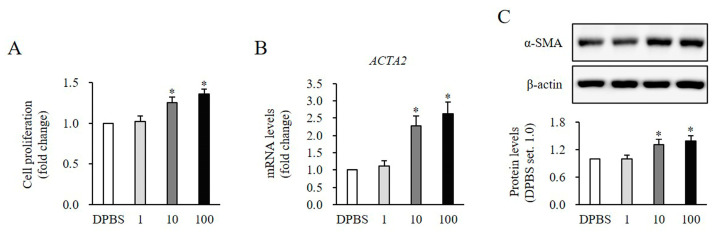
Effects of rSlit1 treatment on the proliferation and differentiation of HNFs. (**A**) Significantly increased proliferation of HNFs was observed following treatment with 10 and 100 ng/mL compared to DPBS-treated cells. (**B**,**C**) Significantly increased levels of both mRNA and protein of α-SMA (*ACTA2*) were observed in HNFs treated with 10 and 100 ng/mL of rSlit1 compared to DPBS-treated cells. DPBS was used as the control. * *p* < 0.05, vs. DPBS. Data represent the mean ± SD; n = 3.

HNFs were treated with rSlit1 at concentrations of 1, 10, and 100 ng/mL for 24 h. Cell proliferation exhibited significant increases at concentrations of 10 and 100 ng/mL in comparison to the DPBS-treated control (1 ng/mL, 1.02 ± 0.07-fold, *p* > 0.05; 10 ng/mL, 1.26 ± 0.06-fold, *p* < 0.05; 100 ng/mL, 1.36 ± 0.06-fold, *p* < 0.05, Figure 2A). Meanwhile, the mRNA expression of the gene encoding differentiation marker α-SMA (*ACTA2*) was significantly increased following rSlit1 treatment (1 ng/mL: 1.11 ± 0.15-fold, *p* > 0.05; 10 ng/mL: 2.29 ± 0.28-fold increase, *p* < 0.05; 100 ng/mL: 2.64 ± 0.33-fold increase, *p* < 0.05; Figure 2B). Similarly, the protein expression of α-SMA was also significantly increased following rSlit1 treatment (1 ng/mL: 1.00 ± 0.08-fold, *p* > 0.05; 10 ng/mL: 1.30 ± 0.12-fold increase, *p* < 0.05; 100 ng/mL: 1.38 ± 0.13-fold increase, *p* < 0.05; Figure 2C). These findings collectively support the notion that rSlit1 enhances the proliferation and differentiation of HNFs.

### 3.3. Recombinant Slit1-Induced Expression of ECM Components in HNFs

**Figure 3:**
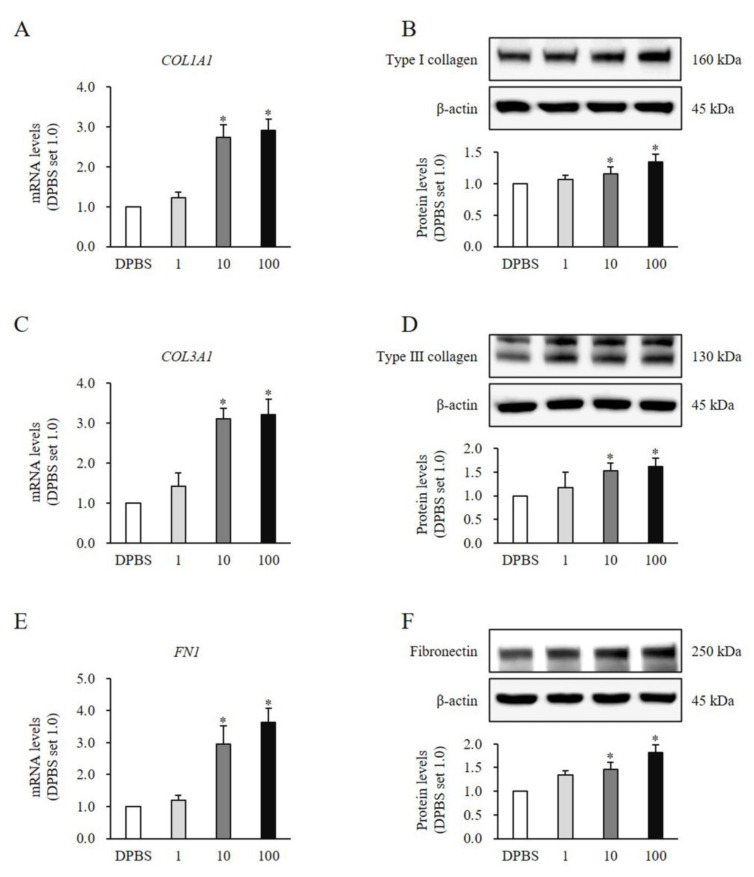
Effects of rSlit1 treatment on the expression of ECM components in HNFs. Significant increases of both mRNA and protein levels of (**A**,**B**) type Ⅰ collagen (*COL1AⅠ*), (**C**,**D**) type Ⅲ collagen (*COL3AⅠ*), and (**E**,**F**) fibronectin (*FN1*) were observed in HNFs treated with 10 and 100 ng/mL of rSlit1 compared to DPBS-treated cells. DPBS was used as the control. * *p* < 0.05, vs. DPBS. Data represent the mean ± SD; n = 3.

The expression of fibrotic markers including type Ⅰ collagen (*COL1AⅠ*) (10 ng/mL, mRNA: 2.73 ± 0.32-fold increase, protein: 1.16 ± 0.11-fold increase, *p* < 0.05; 100 ng/mL, mRNA: 2,90 ± 0.29-fold increase, protein: 1.34 ± 0.13-fold increase, *p* < 0.05; Figure 3A,B), type Ⅲ collagen (*COL3AⅠ*) (10 ng/mL, mRNA: 3.10 ± 0.27-fold increase, protein: 1.53 ± 0.17-fold increase, *p* < 0.05; 100 ng/mL, mRNA: 3.21 ± 0.39-fold increase, protein: 1.61 ± 0.19-fold increase, *p* < 0.05; Figure 3C,D), and fibronectin (*FN1*) (10 ng/mL, mRNA: 2.95 ± 0.57-fold increase, protein: 1.46 ± 0.15-fold increase, *p* < 0.05; 100 ng/mL, mRNA: 3.63 ± 0.43 -fold increase, protein: 1.82 ± 0.17-fold increase, *p* < 0.05; Figure 3E,F) showed significant increases in both mRNA and protein levels in HNFs treated with rSlit1 compared to the DPBS-treated control. Collectively, these results indicated that rSlit1 promotes ECM synthesis in HNFs.

### 3.4. Recombinant Slit1-Induced Changes Related to Epithelial–Mesenchymal Transition (EMT) Phenotype in HNFs

**Figure 4:**
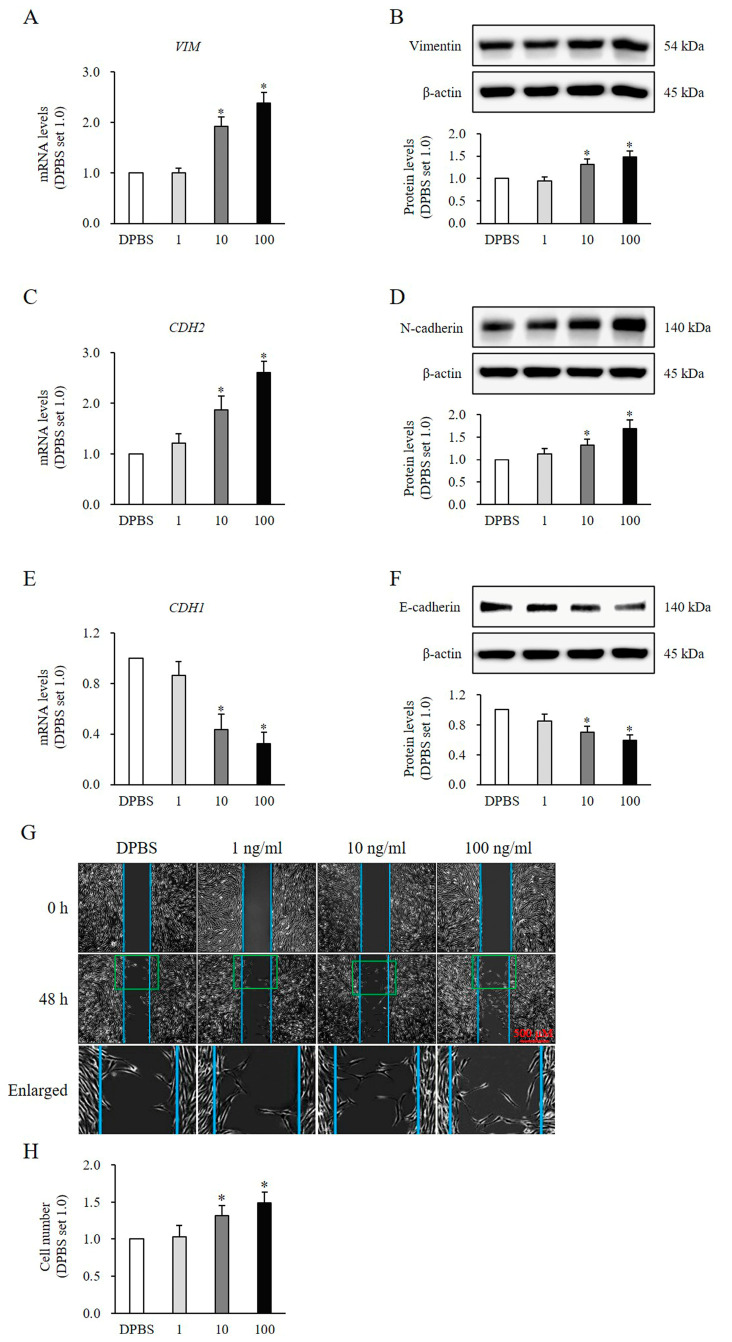
Effects of rSlit1 treatment on the EMT phenotype of HNFs. The mRNA and protein levels exhibited significant increases in the expression of (**A**,**B**) vimentin (*VIM*) and (**C**,**D**) N-cadherin (*CDH2*), whereas a notable decrease in (**E**,**F**) E-cadherin (*CDH1*) expression was observed in HNFs treated with 10 and 100 ng/mL rSlit1, compared to those treated with DPBS. (**G**,**H**) Cell imaging demonstrated enhanced migration of HNFs treated with rSlit1 at concentrations of 10 and 100 ng/mL compared to the DPBS-treated controls. Enlarged images belong to the green box. * *p* < 0.05, vs. DPBS. Data represent the mean ± SD; n = 3.

HNFs treated with rSlit1 for 24 h showed significantly increased expression levels of vimentin (*VIM*) (10 ng/mL, mRNA: 1.92 ± 0.17-fold increase, protein: 1.32 ± 0.11-fold increase, *p* < 0.05; 100 ng/mL, mRNA: 2.38 ± 0.21-fold increase, protein: 1.48 ± 0.13-fold increase, *p* < 0.05; Figure 4A,B) and neural cadherin (N-cadherin) (*CDH2*) (10 ng/mL, mRNA: 1.87 ± 0.18-fold increase, protein: 1.26 ± 0.13-fold increase, *p* < 0.05; 100 ng/mL, mRNA: 2.60 ± 0.22-fold increase, protein: 1.92 ± 0.19-fold increase, *p* < 0.05; Figure 4C,D), while simultaneously demonstrating significant decreases in epithelial cadherin (E-cadherin) (*CDH1*) expression (10 ng/mL, mRNA: 0.44 ± 0.12-fold decrease, protein: 0.70 ± 0.08-fold decrease, *p* < 0.05; 100 ng/mL, mRNA: 0.32 ± 0.09-fold decrease, protein: 0.60 ± 0.07-fold decrease, *p* < 0.05; Figure 4E,F), which all compared to the DPBS-treated control at both the mRNA and protein levels. Furthermore, the migratory capacity of HNFs following rSlit1 treatment for 48 h was significantly enhanced compared to that observed with DPBS (10 ng/mL, 1.31 ± 0.13-fold increase, *p* < 0.05; 100 ng/mL, 1.49 ± 0.14-fold increase, *p* < 0.05; Figure 4G,H). These results further substantiated that rSlit1 promoted the EMT phenotype in HNFs.

### 3.5. Recombinant Slit1-Induced SMAD and Non-SMAD Signaling Pathways in HNFs

**Figure 5:**
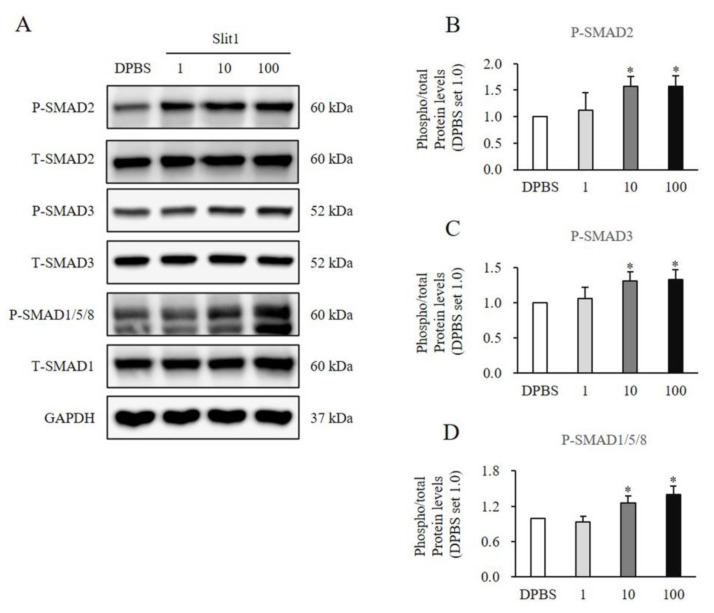
Effects of rSlit1 treatment on expression of SMAD signaling in HNFs. Significantly increased phosphorylated protein expression of (**A**,**B**) SMAD2, (**A**,**C**) SMAD, and (**A**,**D**) SMAD1/5/8 was observed in HNFs treated with 10 and 100 ng/mL rSlit1, compared to DPBS-treated cells. DPBS was used as the control. * *p* < 0.05, vs. DPBS. Data represent the mean ± SD; n = 3.

**Figure 6:**
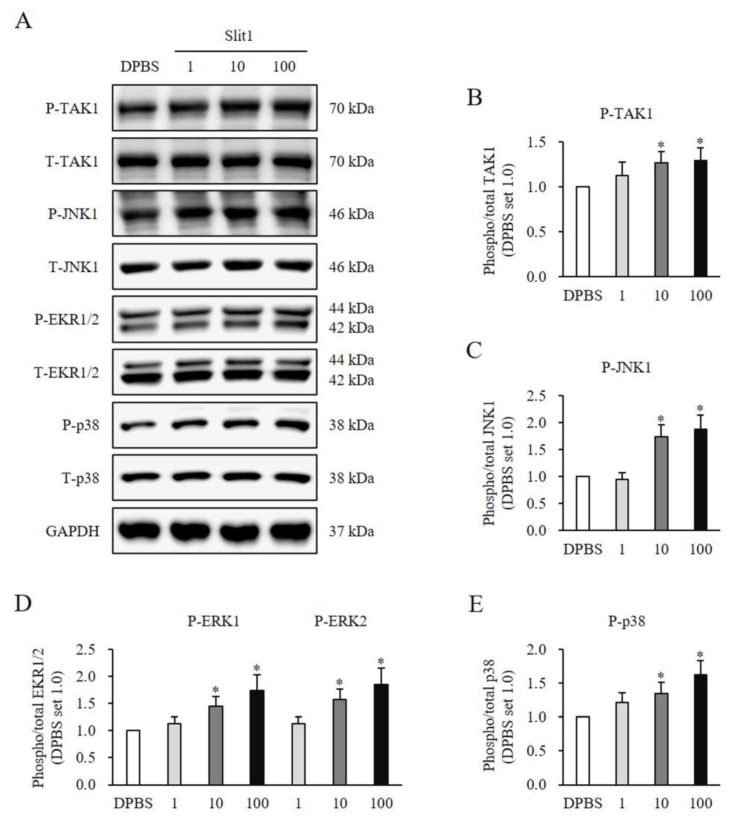
Effects of rSlit1 treatment on expression of non-SMAD signaling in HNFs. Significantly increased phosphorylated protein expression of (**A**,**B**) TAK1, (**A**,**C**) JNK1, (**A**,**D**) ERK1/2, and (**A**,**E**) p38 was observed in HNFs treated with 10 and 100 ng/mL rSlit1 compared to DPBS-treated cells. DPBS was used as the control. * *p* < 0.05, vs. DPBS. Data represent the mean ± SD; n = 3.

HNFs were treated with rSlit1 at concentrations of 1, 10, and 100 ng/mL for 1 h. The expression of SMAD signaling significantly increased in HNFs treated with rSlit1 by elevating protein phosphorylation of SMAD2 (10 ng/mL: 1.57 ± 0.20-fold increase, *p* < 0.05; 100 ng/mL: 1.57 ± 0.20-fold increase, *p* < 0.05; Figure 5A,B), SMAD3 (10 ng/mL: 1.31 ± 0.13-fold increase, *p* < 0.05; 100 ng/mL: 1.33 ± 0.13-fold increase, *p* < 0.05; Figure 5A,C), and SMAD1/5/8 (10 ng/mL: 1.25 ± 0.12-fold increase, *p* < 0.05; 100 ng/mL: 1.40 ± 0.15-fold increase, *p* < 0.05; Figure 5A,D) in comparison to the DPBS-treated control. Additionally, it significantly enhanced non-SMAD signaling by promoting the phosphorylation of TAK1 (10 ng/mL: 1.26 ± 0.13-fold increase, *p* < 0.05; 100 ng/mL: 1.29 ± 0.14-fold increase, *p* < 0.05; Figure 6A,B), JNK1(10 ng/mL: 1.73 ± 0.22-fold increase, *p* < 0.05; 100 ng/mL: 1.88 ± 0.26-fold increase, *p* < 0.05; Figure 6A,C), ERK1/2 (ERK1:10 ng/mL: 1.45 ± 0.18-fold increase, *p* < 0.05; 100 ng/mL: 1.74 ± 0.29-fold increase, *p* < 0.05; ERK2:10 ng/mL: 1.57 ± 0.20-fold increase, *p* < 0.05; 100 ng/mL: 1.85 ± 0.31-fold increase, *p* < 0.05; Figure 6A,D), and p38 (10 ng/mL: 1.35 ± 0.17-fold increase, *p* < 0.05; 100 ng/mL: 1.62 ± 0.21-fold increase, *p* < 0.05; Figure 6A,E) in comparison to the DPBS-treated control. These results indicated that rSlit1 may activate both SMAD and non-SMAD signaling pathways in HNFs.

## 4. Discussion

HTS is a common manifestation of post-burn injuries. Typically, burns that extend into the dermal layer, specifically those classified as second-degree or deeper, result in scar formation [1]. The effective treatment of HTS in the clinical context remains a substantial challenge. Commonly employed treatments encompass surgical removal in combination with compression garments, silicone gel, laser therapy, and intralesional corticosteroid injections. However, these treatments frequently produce less-than-optimal outcomes, sometimes entailing additional surgeries. Pharmacological inhibitors targeting SMAD and non-SMAD signaling pathways, despite showing promising results in vitro and vivo, have not yet been used in patients due to adverse effects observed in clinical trials [14]. Interestingly, elevating vitamin D levels to above 25 ng/mL in patients with HTS could significantly decrease scar width [15]. This therapeutic effect might be ascribed to the activation of nuclear factor erythroid-2-related factor 2 (Nrf2) and p53, along with the inhibition of nuclear factor kappa B (NF-κB) and wingless-related integration site (Wnt)/β-catenin signaling pathways [16]. In our study, we evidenced that Slit1 is a potential target for HTS treatment, providing promising development opportunities for new inhibitors targeting Slit1 or Slit1/Robo signaling.

Slit1 belongs to the Slit family of secreted proteins and was initially identified in the central nervous system (CNS) [9], where it participates in modulating the proliferation and division processes of early neural progenitor cells [17]. Current investigations have demonstrated a correlation between Slits and fibrosis. In normal human liver tissue, the expression of mesenchymal components, including fibroblasts, was observed to predominantly utilize the Slit and Robo gene pool, suggesting the antifibrotic potential of the Slit/Robo pathway [18]. Quantitative RT-PCR showed that the mRNA expression of Slit1 was extremely low in most normal human tissues, including the skin, with the exception of nervous system tissues such as the brain and spinal cord [19]. In this study, we demonstrated that the expression of Slit1 protein in both HTS tissue and extracted HTSFs was significantly elevated compared to that in normal skin tissue and HNFs. To elucidate the role of Slit1 in fibroblasts, we administered rSlit1 to HNF. The results indicate that Slit1 promotes fibroblast proliferation and enhances the expression of αSMA and components of the ECM. These findings suggest that Slit1 may be implicated in HTS formation.

EMT represents a critical event in tissue fibrosis and is characterized by the loss of epithelial traits and the acquisition of mesenchymal traits [20]. Previous qRT-PCR analyses conducted on HTS tissues demonstrated significant upregulation in the expression of snail family transcriptional repressor 2 (slug), twist-related protein 1 (twist1), vimentin, and N-cadherin [21]. In HTSF, the expression of mesenchymal markers such as vimentin, twist1, and N-cadherin was significantly increased, while the expression of the epithelial marker E-cadherin was significantly diminished. This suggests that HTSF exhibits the EMT phenotype [22]. However, fibroblasts possess mesenchymal characteristics and the capacity for independent migration during wound healing [23]. During fibroblast–myofibroblast transition (FMT), the upregulation of αSMA represents a fundamental characteristic, accompanied by the maturation of adhesion complexes. However, this does not correspond to the phenotypic characteristics associated with the EMT [24].

In typical wound healing, keratinocytes are the main participants involved in EMT, undergoing a reversible “partial EMT”. In this state, keratinocytes concurrently express both epithelial and mesenchymal markers, while acquiring a morphological transformation into a migratory phenotype [21]. Comparable phenomena are also observed in epithelial cells and the emergence of a stable epithelial/mesenchymal (E/M) hybrid phenotype that temporarily exhibits mesenchymal characteristics, but it cannot be clarified whether they can fully differentiate into components of fibroblasts/myofibroblast fibrotic foci [25]. However, in the mouse mammary epithelial cell line SCp2 derived from mice, EMT-derived mesenchymal cells may be the primary source of myofibroblasts [26]. During corneal wound healing, normally quiescent stromal keratocytes were demonstrated to transdifferentiate into myofibroblasts following EMT, driven by TGF-β stimulation [27]. Therefore, we hypothesized that the EMT phenotype of HTSF results from the differentiation of EMT-producing keratinocytes into myofibroblasts. Additionally, when normal dermal fibroblasts are treated with exosomes derived from HTSFs, they exhibit enhanced EMT and cell migration, resulting in reduced E-cadherin expression and elevated N-cadherin and vimentin expression [13]. Consequently, we further hypothesized that mesenchymal cells derived from EMT constitute a component of HNF and may continue to transition from partial to complete EMT in vitro.

The cell migration guidance molecules recognized in fibroblasts from the Slit family are limited to Slit2 and Slit3 [28]. However, recent studies have demonstrated that downstream of Slit1/Robo regulates EMT markers such as N-cadherin and E-cadherin, while also being associated with GTPases and β-catenin, which both contribute to cell adhesion [9]. For instance, in glioma cell lines, the knockdown of Slit1 results in the suppression of Wnt/β-catenin signaling pathway, subsequently leading to decreased cell proliferation and adhesion [29]. In the cranial sensory neurons of chickens, Slit1 demonstrates a synergistic interaction with N-cadherin expression [30]. However, Slit1 may occasionally exhibit opposing roles in other tissues. For example, in glioblastoma, the overexpression of Slit1 leads to reduced cell proliferation, adhesion, and viability [31]. In colorectal cancer cells, Slit1 can inhibit both proliferation and migration [32]. Our results demonstrate that rSlit1 enhances the expression of vimentin and N-cadherin, while concurrently reducing the expression of E-cadherin, thereby promoting fibroblast migration. These findings suggest that rSlit1 facilitates EMT and contributes to HTS. In repair of injured human tendons, the elevated expression of N-cadherin can enhance both the migration of fibroblast clusters and the expression of vimentin and αSMA [33].

TGF-β is a potent inducer of fibrosis, with the dysregulation of its signaling pathways implicated in the pathogenesis of tissue fibrosis. For instance, within fibrosing disorders of kidney, heart, and lungs, the overexpressed TGF-β can prominently induce the activation of downstream SMAD signaling and MAPKs in non-SMAD signaling to promote fibrosis [34,35,36]. The activated TAK1 and subsequent MAPKs are also capable of inducing EMT [7]. In HTS tissues, compared to normal tissues, TGF-β expression is markedly elevated and demonstrates prolonged persistence. Additionally, the receptors TβR1 and TβR2 are significantly upregulated [37]. TGF-β-SMAD signaling is served as one of the most extensively investigated potential therapeutic target for HTS [5]. In a previous study, the treatment of HNFs with exosomes from HTSFs resulted in increased phosphorylation of SMAD2 and SMAD1/5/8; TAK1 and its downstream signaling cascades, including p38, ERK, and JNK, exhibited similar increases. Our study demonstrated that rSlit1 exerted a promoting effect in the phosphorylation encompassing of SMAD2, SMAD3, and SMAD1/5/8 within SMAD signaling, and TAK1, JNK, ERK1/2, and p38 within non-SMAD signaling. Slit1 is typically engaged with Robo1, while the secreted protein Slit2 is also capable of binding to the Robo1 receptor [9]. In cardiac and hepatic fibrosis, Slit2/Robo1 signaling significantly upregulates TGF-β1 expression and activates the downstream expression of SMAD2/3 [10,11]. In periodontitis, the overexpression of Slit2 may exacerbate inflammation and immune cell infiltration, potentially activating p38/MAPK through the Slit2/Robo1 signaling pathway [38].

In summary, this study investigated the regulatory role of rSlit1 on TGF-β SMAD and non-SMAD signaling in HNF, as well as its effects on fibrotic and EMT characteristics; however, the directional influence of the EMT on HTS in fibroblasts remains unclear. Our experiments in vitro demonstrated that exogenous Slit1 influences HTS formation; however, additional investigations animal experiment is necessary for future research. In addition, there is undoubtedly the potential that Slit1 can act as a therapeutic target for HTS; however, as of now, specific and effective inhibitors targeting Slit1 are not available.

Presently, a substantial number of treatment strategies on fibrosis targeting TGF-β are in preclinical or clinical stages. Previous research indicated that TGF-β1 is the upstream regulator of Robo1. However, as TGF-β1 imposes an inhibitory effect on the proliferation and migration of breast epithelial cells, Slit/Robo1 plays the same inhibitory role, thereby TGF-β1 is capable of enhancing the effect of Slit1 on cells through upregulating Robo1 [39]. Investigations on cardiac tissue have disclosed that Slit2/Robo1 can promote fibrosis through the SMAD pathway, and Slit2 is positioned upstream of the TGF-β1 signaling pathway [11]. In renal, Slit2/Robo can suppress the transformation of renal fibroblasts into myofibroblasts and the subsequent tissue fibrosis through the inhibition of TGF-β1 and SMAD signaling [40]. During HTS formation, TGF-β1 would exert a positive feedback promotion effect. Firstly, chronic inflammation led to a more persistent secretion of inflammatory growth factors including TGF-β. These TGF-β1 would enhance the production of ECM components and facilitates its autocrine loop, resulting in a higher and higher level of TGF-β1 that further promotes HTS [41]. Based on the existing results, we are incapable of determining whether the elevated TGF-β1 lies upstream or downstream of Slit1/Robo. In our results, the Slit1 crosstalk with TGF-β1. Because they induce each other when cells are treated separately. According to Supplementary Figure S1, rSlit1 significantly increased the expression of TGF-β1. Moreover, downstream signaling pathways of TGF-β are mostly highly complex and encompass organs that sustain human life activities, such as the heart. Consequently, these inhibitory treatment strategies have constantly failed to achieve ideal outcomes in animal experiments or clinical trials [42].

From another perspective, transmembrane protein Robo constitutes the critical passage via which Slit1 actualizes its cellular pathway. We asserted that the inhibitory treatment strategy targeting Slit1 can be established at receptor Robo through direct suppression or engagement in competitive binding. We anticipate that the potential for pursuing the development of Slits inhibitors could be realized in the field of HTS healing in the future.

## 5. Conclusions

We demonstrated that the recombinant Slit1 treatment of normal human fibroblasts enhances EMT and upregulates both SMAD and non-SMAD signaling pathways, ultimately leading to the accumulation of the extracellular matrix. These molecular alterations may facilitate the development of hypertrophic scars. The findings presented herein offer novel perspectives on the underlying pathology and characterize a new potential therapeutic target for post-burn hypertrophic scars: Slit1.

## Figures and Tables

**Table 1:** Demographic characteristics of patients with post-burn hypertrophic scar.

Patients (n = 10)	Age (Years)	Sex	Location of Specimens (Scar/Normal)	Months Post-Burn
1	38	Male	Elbow/elbow	8
2	41	Male	Elbow/elbow	11
3	33	Male	Leg/leg	10
4	36	Male	Thigh/thigh	13
5	42	Male	Chest/chest	12
6	29	Male	Chest/chest	13
7	44	Male	Scalp/scalp	16
8	33	Male	Thigh/thigh	19
9	29	Male	Abdomen/abdomen	13
10	29	Female	Face/scalp	15
11	27	Female	Arm/arm	11
12	38	Female	Shoulder/shoulder	9
13	43	Female	Hand/hand	7

**Table 2:** Real-time PCR primer sequences.

Gene	Forward (5′ → 3′)	Reverse (5′ → 3′)
*SLIT1*	CTCCTTCACCAACATGAGCCAG	AGGGTGGAGATGTCATTGCCGT
*ACTA2*	CCGACCGAATGCAGAAGGA	ACAGAGTATTTGCGCTCCGAA
*FN1*	CCAGTCCACAGCTATTCCTG	ACAACCACGGATGAGCTG
*COL1A1*	ATGTTCAGCTTTGTGGACCTC	CTGTACGCAGGTGATTGGTG
*COL3A1*	CACTGGGGAATGGAGCAAAAC	ATCAGGACCACCAATGTCATAGG
*CDH* *1*	CCAGTCCACAGCTATTCCTG	ACAACCACGGATGAGCTG
*CDH2*	ACCGACACTCCTACAAGATTT	GCAGAAACAAGTTGGTTGGATA
*VIM*	GTCAGAACTAAAGGAGCTGC	TGTTGCTGTCCAAGTTGCTC
*GAPDH*	CATGAGAAGTATGACAACAGCCT	AGTCCTTCCACGATACCAAAGT

**Table 3:** Primary antibodies used in Western blot analysis.

Target	Host	Dilution	Company (Cat. No.)
GAPDH	Rabbit	1:1000	Cell Signaling Technology (2118S)
GAPDH	Mouse	1:1000	Santa Cruz Technology (sc-47724)
β-Actin	Rabbit	1:2000	Cell Signaling Technology (4967S)
β-Actin	Mouse	1:1000	Santa Cruz Biotechnology (sc-1616)
Slit1	Mouse	1:400	Santa Cruz Biotechnology (sc-376756)
α-SMA	Mouse	1:500	Abcam (ab7817)
Fibronectin	Rabbit	1:2000	Abcam (ab6328)
Type I collagen	Rabbit	1:1000	Abcam (ab34710)
Type III collagen	Rabbit	1:1000	Abcam (ab7778)
Vimentin	Mouse	1:3000	Abcam (ab92547)
N-Cadherin	Mouse	1:1000	Invitrogen (333900)
E-Cadherin	Mouse	1:1000	Abcam (ab76055)
Phospho-SMAD2	Rabbit	1:1000	Cell Signaling Technology (3108S)
SMAD2	Rabbit	1:1000	Abcam (ab33875)
Phospho-SMAD3	Rabbit	1:1000	Invitrogen (MA5-14936)
SMAD3	Rabbit	1:1000	Cell Signaling Technology (9523S)
Phospho-SMAD1/5/8	Rabbit	1:1000	Merck Millipore (AB3848)
SMAD1	Rabbit	1:1000	Cell Signaling Technology (6944S)
Phospho-TAK1	Rabbit	1:1000	Cell Signaling Technology (9339S)
TAK1	Rabbit	1:1000	Cell Signaling Technology (5206S)
Phospho-p38	Mouse	1:1000	Cell Signaling Technology (9216S)
p38	Rabbit	1:1000	Cell Signaling Technology (8690S)
Phospho-JNK	Rabbit	1:1000	Cell Signaling Technology (9251S)
JNK	Rabbit	1:1000	Cell Signaling Technology (9252S)
Phospho-ERK	Rabbit	1:1000	Cell Signaling Technology (4370S)
ERK	Mouse	1:1000	Cell Signaling Technology (4696S)

## Data Availability

For inquiries regarding access to the datasets generated and/or analyzed in this study, please directly correspond to the corresponding author.

## Supplementary Materials

Following supporting information can be downloaded at: https://www.mdpi.com/article/10.3390/medicina60122051/s1, Figure S1: rSlit1 treatment induced protein expression of TGF-β1 in HNFs.

## References

[1] Pradhan M., Pethe P. (2023). The Molecular Mechanisms Involved in the Hypertrophic Scars Post-Burn Injury. Yale J. Biol. Med..

[2] Bharadia S.K., Burnett L., Gabriel V. (2023). Hypertrophic Scar. Phys. Med. Rehabil. Clin. N. Am..

[3] Schuster R., Younesi F., Ezzo M., Hinz B. (2023). The Role of Myofibroblasts in Physiological and Pathological Tissue Repair. Cold Spring Harb. Perspect. Biol..

[4] Moretti L., Stalfort J., Barker T.H., Abebayehu D. (2022). The interplay of fibroblasts, the extracellular matrix, and inflammation in scar formation. J. Biol. Chem..

[5] Zhang T., Wang X.F., Wang Z.C., Lou D., Fang Q.Q., Hu Y.Y., Zhao W.Y., Zhang L.Y., Wu L.H., Tan W.Q. (2020). Current potential therapeutic strategies targeting the TGF-β/Smad signaling pathway to attenuate keloid and hypertrophic scar formation. Biomed. Pharmacother..

[6] Deng Z., Fan T., Xiao C., Tian H., Zheng Y., Li C., He J. (2024). TGF-β signaling in health, disease, and therapeutics. Signal Transduct. Target. Ther..

[7] Zhang Y.E. (2017). Non-Smad Signaling Pathways of the TGF-β Family. Cold Spring Harb. Perspect. Biol..

[8] DesJardins-Park H.E., Gurtner G.C., Wan D.C., Longaker M.T. (2022). From Chronic Wounds to Scarring: The Growing Health Care Burden of Under- and Over-Healing Wounds. Adv. Wound Care (New Rochelle).

[9] Tong M., Jun T., Nie Y., Hao J., Fan D. (2019). The Role of the Slit/Robo Signaling Pathway. J. Cancer.

[10] Chang J., Lan T., Li C., Ji X., Zheng L., Gou H., Ou Y., Wu T., Qi C., Zhang Q. (2015). Activation of Slit2-Robo1 signaling promotes liver fibrosis. J. Hepatol..

[11] Liu Y., Yin Z., Xu X., Liu C., Duan X., Song Q., Tuo Y., Wang C., Yang J., Yin S. (2021). Crosstalk between the activated Slit2-Robo1 pathway and TGF-β1 signalling promotes cardiac fibrosis. ESC Heart Fail..

[12] Gong L., Si M.S. (2023). SLIT3-mediated fibroblast signaling: A promising target for antifibrotic therapies. Am. J. Physiol. Heart Circ. Physiol..

[13] Cui H.S., Kim D.H., Joo S.Y., Cho Y.S., Kim J.B., Seo C.H. (2022). Exosomes derived from human hypertrophic scar fibroblasts induces smad and TAK1 signaling in normal dermal fibroblasts. Arch. Biochem. Biophys..

[14] Finnson K.W., Almadani Y., Philip A. (2020). Non-canonical (non-SMAD2/3) TGF-β signaling in fibrosis: Mechanisms and targets. Semin. Cell Dev. Biol..

[15] Ince B., Uyar I., Dadaci M. (2019). Effect of Vitamin D Deficiency on Hypertrophic Scarring. Dermatol. Surg. Off. Publ. Am. Soc. Dermatol. Surg..

[16] Slominski A.T., Kim T.K., Janjetovic Z., Slominski R.M., Li W., Jetten A.M., Indra A.K., Mason R.S., Tuckey R.C. (2024). Biological Effects of CYP11A1-Derived Vitamin D and Lumisterol Metabolites in the Skin. J. Investig. Dermatol..

[17] Borrell V., Cárdenas A., Ciceri G., Galcerán J., Flames N., Pla R., Nóbrega-Pereira S., García-Frigola C., Peregrín S., Zhao Z. (2012). Slit/Robo signaling modulates the proliferation of central nervous system progenitors. Neuron.

[18] Basha S., Jin-Smith B., Sun C., Pi L. (2023). The SLIT/ROBO Pathway in Liver Fibrosis and Cancer. Biomolecules.

[19] Dickinson R.E., Dallol A., Bieche I., Krex D., Morton D., Maher E.R., Latif F. (2004). Epigenetic inactivation of SLIT3 and SLIT1 genes in human cancers. Br. J. Cancer.

[20] Stone R.C., Pastar I., Ojeh N., Chen V., Liu S., Garzon K.I., Tomic-Canic M. (2016). Epithelial-mesenchymal transition in tissue repair and fibrosis. Cell Tissue Res..

[21] Yuan F.L., Sun Z.L., Feng Y., Liu S.Y., Du Y., Yu S., Yang M.L., Lv G.Z. (2019). Epithelial-mesenchymal transition in the formation of hypertrophic scars and keloids. J. Cell Physiol..

[22] Cui H.S., Hong A.R., Kim J.B., Yu J.H., Cho Y.S., Joo S.Y., Seo C.H. (2018). Extracorporeal Shock Wave Therapy Alters the Expression of Fibrosis-Related Molecules in Fibroblast Derived from Human Hypertrophic Scar. Int. J. Mol. Sci..

[23] Trepat X., Chen Z., Jacobson K. (2012). Cell migration. Compr. Physiol..

[24] D’Urso M., Kurniawan N.A. (2020). Mechanical and Physical Regulation of Fibroblast-Myofibroblast Transition: From Cellular Mechanoresponse to Tissue Pathology. Front. Bioeng. Biotechnol..

[25] Jolly M.K., Ward C., Eapen M.S., Myers S., Hallgren O., Levine H., Sohal S.S. (2018). Epithelial-mesenchymal transition, a spectrum of states: Role in lung development, homeostasis, and disease. Dev. Dyn..

[26] Radisky D.C., Kenny P.A., Bissell M.J. (2007). Fibrosis and cancer: Do myofibroblasts come also from epithelial cells via EMT?. J. Cell Biochem..

[27] Shu D.Y., Lovicu F.J. (2017). Myofibroblast transdifferentiation: The dark force in ocular wound healing and fibrosis. Prog. Retin. Eye Res..

[28] Chang H.Y., Chi J.T., Dudoit S., Bondre C., van de Rijn M., Botstein D., Brown P.O. (2002). Diversity, topographic differentiation, and positional memory in human fibroblasts. Proc. Natl. Acad. Sci. USA.

[29] Zheng Y., Xiao M., Zhang J., Chang F. (2022). Micro RNA-640 Targeting SLIT1 Enhances Glioma Radiosensitivity by Restraining the Activation of Wnt/β-Catenin Signaling Pathway. Br. J. Biomed. Sci..

[30] Shiau C.E., Bronner-Fraser M. (2009). N-cadherin acts in concert with Slit1-Robo2 signaling in regulating aggregation of placode-derived cranial sensory neurons. Development.

[31] Luo C., Lu Z., Chen Y., Chen X., Liu N., Chen J., Dong S. (2021). MicroRNA-640 promotes cell proliferation and adhesion in glioblastoma by targeting Slit guidance ligand 1. Oncol. Lett..

[32] Shuai W., Wu J., Chen S., Liu R., Ye Z., Kuang C., Fu X., Wang G., Li Y., Peng Q. (2018). SUV39H2 promotes colorectal cancer proliferation and metastasis via tri-methylation of the SLIT1 promoter. Cancer Lett..

[33] Zhuang Y., Lin F., Xiang L., Cai Z., Wang F., Cui W. (2024). Prevented Cell Clusters’ Migration Via Microdot Biomaterials for Inhibiting Scar Adhesion. Adv. Mater..

[34] Lan H.Y. (2011). Diverse roles of TGF-β/Smads in renal fibrosis and inflammation. Int. J. Biol. Sci..

[35] Frangogiannis N.G. (2022). Transforming growth factor-β in myocardial disease. Nat. Rev. Cardiol..

[36] Ji Y., Dou Y.N., Zhao Q.W., Zhang J.Z., Yang Y., Wang T., Xia Y.F., Dai Y., Wei Z.F. (2016). Paeoniflorin suppresses TGF-β mediated epithelial-mesenchymal transition in pulmonary fibrosis through a Smad-dependent pathway. Acta Pharmacol. Sin..

[37] Kiritsi D., Nyström A. (2018). The role of TGFβ in wound healing pathologies. Mech. Ageing Dev..

[38] Wang L., Zheng J., Pathak J.L., Chen Y., Liang D., Yang L., Sun H., Zhong M., Wu L., Li L. (2020). SLIT2 Overexpression in Periodontitis Intensifies Inflammation and Alveolar Bone Loss, Possibly via the Activation of MAPK Pathway. Front. Cell Dev. Biol..

[39] Macias H., Moran A., Samara Y., Moreno M., Compton J.E., Harburg G., Strickland P., Hinck L. (2011). SLIT/ROBO1 signaling suppresses mammary branching morphogenesis by limiting basal cell number. Dev. Cell.

[40] Yuen D.A., Huang Y.W., Liu G.Y., Patel S., Fang F., Zhou J., Thai K., Sidiqi A., Szeto S.G., Chan L. (2016). Recombinant N-Terminal Slit2 Inhibits TGF-β-Induced Fibroblast Activation and Renal Fibrosis. J. Am. Soc. Nephrol. JASN.

[41] Ogawa R. (2017). Keloid and Hypertrophic Scars Are the Result of Chronic Inflammation in the Reticular Dermis. Int. J. Mol. Sci..

[42] Peng D., Fu M., Wang M., Wei Y., Wei X. (2022). Targeting TGF-β signal transduction for fibrosis and cancer therapy. Mol. Cancer.

