# Supplementary material for: Slit1 Promotes Hypertrophic Scar Formation Through the TGF-β Signaling Pathway
Source: Medicina (Kaunas). 2024 Dec 12;60(12):2051. doi: 10.3390/medicina60122051 (PMC11678377; doi:10.3390/medicina60122051)
Supplement: Supplementary file 1 [file medicina-60-02051-s001.zip › medicina-3343081-supplementary.pdf]

Recombinant Slit1 induced protein expression of TGF-β1 in HNFs

The protein expression of TGF-β1 in human normal fibroblasts treated with rSlit1 was significantly higher compared with the control group (10 ng/mL: 1.41±0.16-fold increase, $P < 0.05$; 100 ng/mL: 1.38±0.08-fold increase, $P < 0.05$).

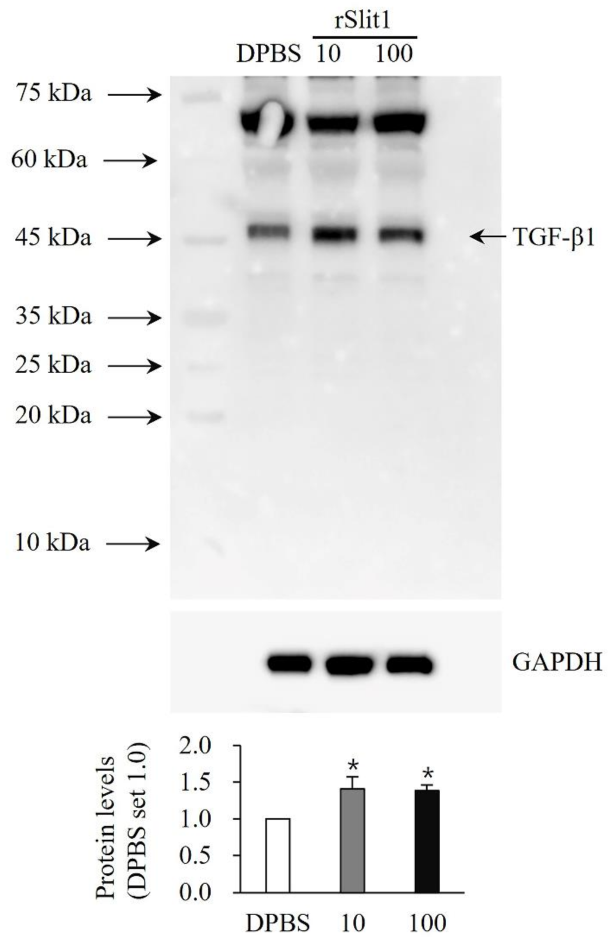

Supplementary figure 1. rSlit1 treatment induced protein expression of TGF-β1 in HNFs. Significantly increases of protein levels of TGF-β1 was observed in HNFs treated with 10 and 100 ng/mL of rSlit1 compared to DPBS-treated cells. DPBS was used as the control. *$P < 0.05$, vs DPBS. Data represents the mean ± SD; n = 3.
